# Efficient option of industrial wastewater resources in cement mortar application with river-sand by microbial induced calcium carbonate precipitation

**DOI:** 10.1038/s41598-020-62666-9

**Published:** 2020-04-21

**Authors:** Yi-Hsun Huang, How-Ji Chen, Jyoti Prakash Maity, Chien-Cheng Chen, An- Cheng Sun, Chien-Yen Chen

**Affiliations:** 1 Department of Civil Engineering, National Chung-Hsing University, Taichung, 402 Taiwan; 2 Department of Earth and Environmental Sciences, National Chung Cheng University, 168 University Road, Ming-Shung, Chiayi County 62102 Taiwan; 3 School of Civil Engineering and Surveying and International Centre for Applied Climate Science, University of Southern Queensland, Toowoomba, Australia; 4 Department of Biotechnology, National Kaohsiung Normal University, Kaohsiung, 82444 Taiwan; 5 Department of Chemical Engineering and Materials Science, Yuan-Ze University, 135 Yuan-Tung Road, Chung-Li, 32003 Taiwan; 6 Center for Innovative Research on Aging Society, AIM-HI, National Chung Cheng University, 168, University Rd., Min-Hsiung, Chiayi 62102 Taiwan

**Keywords:** Pollution remediation, Biomineralization

## Abstract

The industrial wastewater disposal has been growing attention for environmental protection and resource substitution, current decades. Similarly, the durability enhancement of concrete has increased attention by microbial induced CaCO_3_ precipitation (MICP) process (biocalcification). However, ecofriendly utilization of industrial wastewater in concrete formation is unstudied so far. The present study was carried out to evaluate the effect of industrial wastewater on the formation of cement mortar, compressive strength and water absorption. The biocement mortar strength (y) increased (y = 0.5295×^2^ + 1.6019×+251.05; R^2^ = 0.9825) with increasing percentage of organic wastewater (x) (BM_0_ – BM_100_) by MICP, where highest strength (280.75 kgf/cm^2^) was observed on BM_100_ (100% wastewater), compared to control (252.05 kgf/cm^2^). The water absorption (y) of biocement mortar decreases (y = −0.0251×^2^–0.103× + 15.965; R^2 ^= 0.9594) with increment of wastewater (x) (%) (BM_0_ – BM_100_), where a minimum-water-absorption (14.42%) observed on BM_100_, compared to control (15.89%). SEM micrograph and XRD shows the formation of most-distinctive CaCO_3_ crystallization (aragonite/calcite) (acicular, brick shape, massive and stacked structure) inside biocement mortar (BM_100_), which fills the pores within cement mortar to form a denser structure, by microbial organic wastewater. Thus, present findings implied a cost-effective of MICP technology to improve the concrete properties along with the mitigation of industrial wastewater pollution, which goes some way towards solving the problem of industrial wastewater pollution.

## Introduction

Since the industrial revolution in ^18^th century, the quality of life of human has improved drastically and the population grows exponentially, worldwide. As a result, the demand for sturdy and comfortable houses has been on the rise. The cement, a most common building material that use for constriction, worldwide. However, manufacturing cement produces a large amount of carbon dioxide that harms the environment^1,2^. Therefore, the researchers in civil engineering sector have been investigating to extend the service life of cement in building constriction^3–6^. On the other hand, the researcher reported that the bioremediation process is able to strengthen the structural integrity of buildings and reduces water absorption. Furthermore, the researchers have been trying to report, how organisms repair themselves, and they have been trying to develop ecofriendly bioremediation systems^7–9^. However, the bioremediation along with repair system has not been reported clearly, so far.

In natural environment, small sand grains solidify to become sandstone under biogeochemical process (microorganisms, time, and pressure). At adequate calcium concentration, the calcium reacts with carbonate ions to precipitate calcium carbonate that acting as a gluing agent, where the calcium carbonate helps the transformation process of sand solidification. In this process, the microorganisms metabolize and produce urease that transform the urea into ammonium and carbonate ions^10,11^. This bio-mineralization occurs by microbial induce calcium participation, (MICP) process^12–15^.

The industrial wastewater is one of the complications to ecofriendly progress in human civilization. Carelessly discharging the wastewater into water bodies are affecting the physical, chemical, and biological changes to the environment, since it (wastewater) is not only harmful to the environment but also to human health. Therefore, industrial sectors are obligated to install comprehensive wastewater processing/treatment system. Such system often requires proper equipment, ecofriendly technology, as well as funding for wastewater treatment to permissible limit, that often increase the production cost^16,17^. However, the ecofriendly wastewater treatment is a great challenge in present-day research. Thus, it is an urgent need to utilize of wastewater for beneficial purpose of ecofriendly environment pollution management. The investigator utilized the urase enzyme of urealytic microorganism (example: *Sporosarcina pasteurii* and *Bacillus sphaericus*) for MICP process, through which the mineral can precipitate^18–22^. The organic wastewater of food industries, contains microbial nutrients and a mass amount of microorganism (Example: bacteria), may produces urease in wastewater^23^. Thus, the urease containing wastewater can be used for MICP process by the possible replacement of the aforementioned microorganism/bacteria that may benefits of ecofriendly utilization of industrial wastewater with cost minimization of industrial product^23^. Furthermore, the utilization of industrial wastewater could reduce the cost for wastewater processing in industries where, the main problem of MICP has always been high-cost.

Considering the background, the present study focuses on the utilization organic wastewater for MICP process to produce biocement mortar. The wastewater of food industry was used to produce biocement mortar. In addition, the mechanical properties such as strength and water absorption, as well as the physiochemical properties of the biocement mortar by SEM and XRD, were taken under study.

## Results and Discussion

### Microbiologically induced calcite precipitation by industrial wastewater

#### Microbiologically induced calcite precipitation and characterization of industrial wastewater

**Figure 1:**
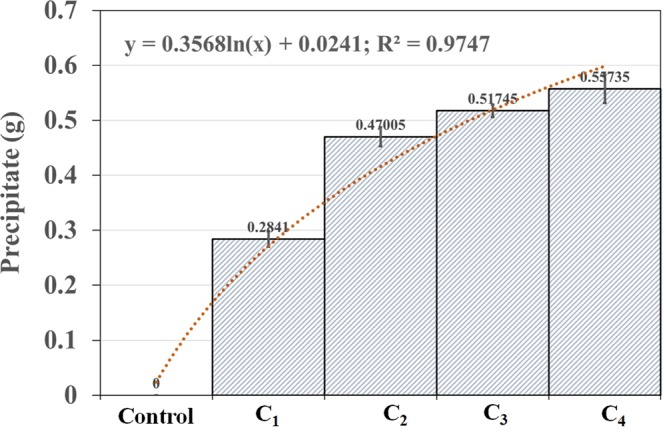
Microbiologically induced calcite precipitation by industrial wastewater in different experimental conditions (C_1_ – C_4_) (Table 2). Bar diagram represents mean ± SD, where n = 3.

The basic characteristics of industrial water is shown in Table 1. The industrial wastewater was noticed slightly higher pH at 8.40 ± 0.5. COD of wastewater was found significantly high as 1200.10 ± 2.5 mg/l compare to BOD (10.50 ± 0.12 mg/l). This is indicated that the higher amount of oxygen required to chemically oxidize organic compounds compare to the amount of oxygen required to biologically oxidize the organics in the industrial wastewater. The temperature of wastewater was observed 30.90 ± 0.89 °C. The CFU of wastewater was noticed as 10^3^ to 10^5^ (cfu/ml), where as the urease activity was observed as 0.894 ± 0.01 (mol/l). The results of the varied parameters of urea and Ca(NO_3_)_2_ with 40 ml of wastewater in different experimental conditions (C_1_-C_4_) (Table 2) shows that the precipitation was increased with the increasing urea and Ca(NO_3_)_2_ concentration, significantly (y = 0.3568ln(x) + 0.0241; R² = 0.9747) (Fig. 1). The precipitation was not occurred in control condition due to absence of urea and Ca(NO_3_)_2_. Thus, the urea and Ca(NO_3_)_2_ are essential for microbiologically induced calcite precipitation in wastewater, which are very active in precipitation process. Hammes *et al*.^24^, reported that the strain-specific calcification occurred during ureolytic microbial carbonate precipitation by *Bacillus sphaericus*. In another study, the Ca^2+^ remove from industrial wastewater by MICP process through ureolytic microorganisms^25^. The precipitate was reported as CaCO_3_ which are composed of predominantly calcite crystals with little vaterite crystals by *Sporosarcina pasteurii* strain ATCC 11859^26^. In the present study, particularly, a rapid precipitation (0.47 g) was occurred within the concentration of 0.3 M of Ca(NO_3_)_2_, where a maximum precipitation was observed as 0.55 g at concentration of 1.1 M of Ca(NO_3_)_2_. Therefore, the microbial community in industrial wastewater is effective to precipitate the calcium mineral, naturally.

**Table 1:** Basic parameter of industrial wastewater. Data represents mean ± SE.

Parameter	Concentration in water	Guideline/Standard (MOEAIDB)^39^
pH	8.40 ± 0.5	6–9
COD (mg/l)	1200.10 ± 2.5	100
BOD (mg/l)	10.50 ± 0.12	30
Color (Pt-Co)	51.00 ± 0.05	550
TSS (mg/l)	16.10 ± 0.08	30
Temp (°C)	30.90 ± 0.89	35
CFU (cfu/ml)	10^3^–10^5^	—
Urease activity (mol/l)	0.894 ± 0.01	—

**Table 2:** The varied parameters of urea and Ca(NO_3_)_2_ with 40 ml of wastewater in different experimental conditions (C_1_ – C_4_).

Chemical/wastewater	Control	C_1_	C_2_	C_3_	C_4_
Wastewater (ml)	40	40	40	40	40
Urea (M)	0	1.1	1.1	1.1	1.1
Ca(NO_3_)_2_ (M)	0	0.1	0.3	0.5	1.1

#### Characterization of synthesized material by microbiologically induced calcite precipitation

**Figure 2:**
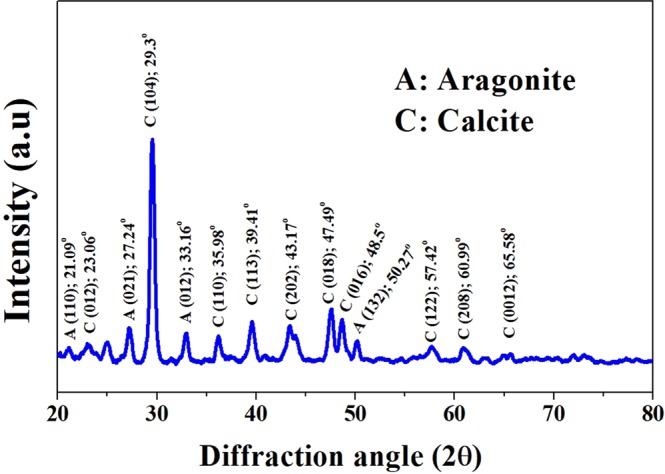
XRD spectrum of synthesized material (C_4_ is the best one) by microbiologically induced calcite precipitation.

The XRD of the material, synthesized by microbial community of wastewater are shown in Fig. 2. The results indicate that the peak intensities (at *2θ*) are observed at 23.06°, 29.3°, 35.98°, 39.41°, 43.17°, 47.49°, 48.5°, 57.42°, 60.99°, and 65.58°, that representing the Miller indices of calcite phase at (012), (104), (110), (113), (202), (018), (016), (122), (208) and (0012), respectively. The peak intensity at 21.09°, 27.24°, 33.16° and 50.27° was observed, which represent the aragonite mineral phases at Miller index of (110), (021), (012), and (132), respectively. The calcite and aragonite are stable forms of calcium carbonate. The present results are comparable with the findings of Torres *et al*.^27^ (2013), where the calcite and vaterite are precipitated in different proportions and shapes by several microorganisms in domestic wastewater. Thus, it is confirming that the calcium carbonate precipitation occurs by microbial community of wastewater by MICP process.

### Formation and characterization of biocement mortar

#### Formation of biocement mortar and compressive strength

**Figure 3:**
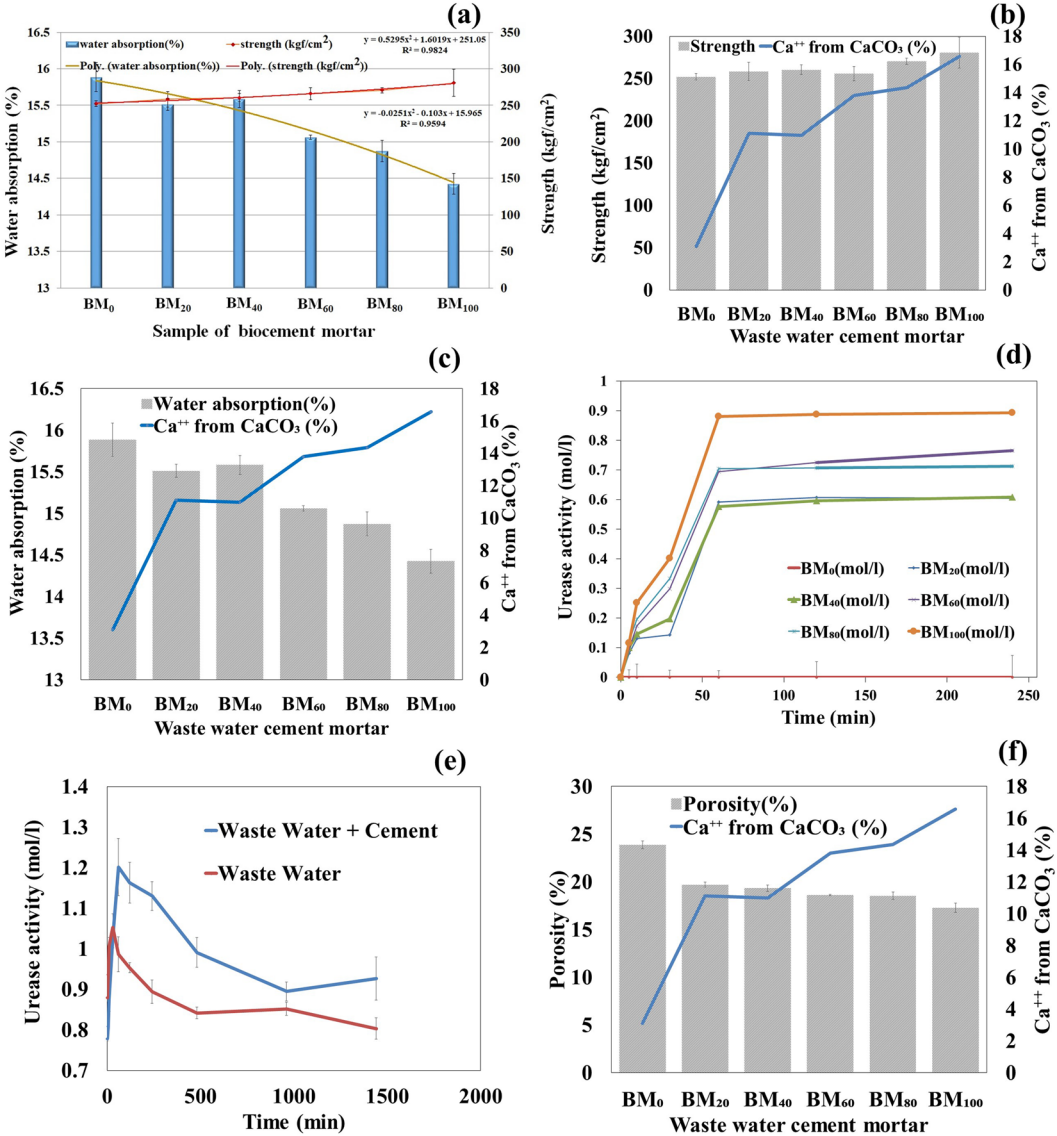
(**a**) The compressive strength and water absorption of biocement mortar in different samples treated with different percentage (%) of wastewater (BM_0_, BM_20_, BM_40_, BM_60_, BM_80_ and BM_100_). Line and Bar diagram represents mean ± SD, where n = 3. The line of polynomial relationship in between wastewater percentage (%) vs compressive strength and wastewater percentage (%) water absorption of biocement mortar. (**b**) Change of compressive strength due to CaCO_3_ formation in different wastewater cement mortar. (**c**) Change of water absorption due to CaCO_3_ formation in different wastewater cement mortar. (**d**) Urease activity of different percentage (%) of wastewater with different time in biocement mortar. (**e**) Urease activity of wastewater+cement and waste water in different time. (**f**) Porosity vs CaCO_3_ formation in different percentage (%) of wastewater (BM_0_, BM_20_, BM_40_, BM_60_, BM_80_ and BM_100_).

The organic wastewater, which contain microbial consortium that influences on water adsorption rate and compressive strength of biocement mortar (Fig. 3a–d). The study highlights the effect of organic wastewater on the compressive strength of cement mortar. The effect of organic waste water on compressive strength of cement mortar are shown in Fig. 3a–b. The compressive strength of cement mortar without organic wastewater was 252.05 kgf/cm^2^ at 28 days, where the compressive strength observed as 258.36 kgf/cm2, 260.44 kgf/cm^2^, 265.89 kgf/cm^2^, 270.65 kgf/cm^2^ and 280.75 kgf/cm^2^ in the treated group of 20%, 40%, 60%, 80% and 100% organic wastewater, respectively at 28days incubation. The polynomial relationship (y = 0.5295×^2^ + 1.6019×+ 251.05) was observed between compressive strength and increment of wastewater percentage (%) (BM_0_, BM_20_, BM_40_, BM_60_, BM_80_ and BM_100_). The regression analysis in between independent variable as wastewater percentage (x) and dependent variable compressive strength (y) reflects a positive polynomial relation (R^2^ = 0.9825) (i.e. compressive strength increases with the increasing of wastewater percentage). The cement mortar treated with 100% wastewater was observed the highest strength (280.75 kgf/cm^2^) compared to control after 28 days. Therefore, the strength of biocement mortar and CaCO_3_ precipitation were increased with the increasing amount of organic wastewater (Fig. 3a,b). The current findings of compressive strength are comparable with the finding of Chahal *et al*.^18,28^, where researchers were indicated the compressive strength was increased in presence of microorganism (*S. pasteurii*). The optimum compressive strength was reported 10^5^ cells/ml, whereas the matrix integrity disrupts due to excessive bacterial activity at 10^7^ cells/ml^18,28^. In another research^29^, the compressive strength of bacterial concrete was reported to be increased in 10^3^–10^5^ (cfu/ml), whereas the strength was found to be decreased in/after 10^7^ (cfu/ml), compared to the concrete sample without bacteria. In the present study, 10–10^3^ (cfu/ml) of bacteria are survived after mixing with cement, which produced the urease enzyme. It is confirming that the reason may be that urease enzyme (produced by bacteria in organic wastewater in Table 1) reacts with urea and calcium nitrate which can produce calcium carbonate precipitation (see XRD of material in previous section)^22^. The cement mortar provides additional pores (during hydration reaction), where the calcium carbonate is predicated and fully filled the porosity of biocement mortar. Although the chemical substances contained in organic wastewater which decreases the binding rate of calcium and citrate in hydration reaction as well as produces a retarding effect. However, in addition of organic wastewater totally (100%) that can effectively improve the strength of cement mortar compare to control.

#### Formation of biocement mortar and water absorption

Similar to the compressive strength, the organic wastewater influences on the water absorption capacity of biocement mortar formation. The effects of organic wastewater on water absorption in cement mortar are shown in Fig. 3. The water absorption of biocement mortar was noticed as 15.89% at 28 days without organic wastewater, whereas 15.51%, 15.58%, 15.06% and 14.87% of water absorption in biocement mortar were observed in the treated group of 20%, 40%, 60% and 80% organic waste water, respectively at 28days. The biocement mortar treated with 100% wastewater was observed a water absorption of 14.42% after 28 days. A polynomial relationship (y = −0.0251×^2^–0.103×+ 15.965) was observed between water absorption and increment of wastewater percentage (%) (BM_0_, BM_20_, BM_40_, BM_60_, BM_80_ and BM_100_). The regression analysis in between independent variable as wastewater percentage (x) and dependent variable water absorption (y) reflects a negative polynomial relation (R^2^ = 0.9594) (i.e. water absorption decreases with the increasing of wastewater percentage). Thus, the water absorption decreases as the proportion of wastewater increases in the treatment process of biocement mortar formation. Chahal *et al*.^28^ observed a four-times reduction of water absorption in fly ash concrete with 10^5^ cells/ml of *S. pasteurii*. In another study, Chahal *et al*.^18^ reported a maximum reduction of water absorption with 10^5^ cells/ml for 10% silica fume concrete at 91 days; however, concrete with 5% silica fume gave 0.1% water absorption (minimum) at 91 days, which was 0.3% at 28 days. The waterproofing effect was reported to increase with increasing calcium dosages in the presence of *Bacillus sphaericus* LMG 225 57, whereas for a while the calcium dosage of 17 g Ca^2+^ m^−2^ the water absorption was reported similar to that of untreated cases. in a 50% decrease of the rate of water absorption was reported at a concentrations of 67 g Ca^2+^ m^−2^ ^7^. In another report, the surface deposition of calcium carbonate crystals decreased the water absorption from 65% to 90% depending on the porosity of the material by *B. sphaericus*^30^. The ureolytic bacteria such as *Bacillus sphaericus* are able to precipitate CaCO_3_ in their micro-environment by conversion of urea into ammonium and carbonate. Thermogravimetric analysis showed that bacteria were able to precipitate CaCO_3_ crystals inside the cracks, as a result the permeability of the biocement mortar decreased^31^. In present study shows that the urease activity plays an important role of the CaCO_3_ formation. The urease activity (mol/l) was observed in wastewater, which was increased significantly upto 60 min; however, the activity was decreased a bit with the decreasing concentration of wastewater (Fig. 3d). On the other hand, the urease activity was noticed higher in the mixer of cement with wastewater, compare to only waste water (Fig. 3e). Therefore, urease activity helps to precipitate the calcium carbonate to the mixture of biocement mortar. The water absorption decreased with the increasing of wastewater concentration or CaCO_3_ formation (Fig. 3c). Thus, these results reflect the formation and precipitation of calcium carbonate from urea and calcium nitrate in presence of urease from bacteria in organic wastewater. The Fig. 3f shows that the porosity of the biocement mortar decreases with the CaCO_3_ precipitation and it is confirmed that the precipitated calcium carbonate effectively fills pores on and within (inside) the surface of the biocement mortar. Therefore, the investigation documents the calcium carbonate precipitation as a result reduction of water absorption on and within biocement mortar, which provides a hopeful solution for durability of cement. Furthermore, the precipitation of calcium carbonate could also fill the pores inside the cement mortar which increases the density and structural strength of the cement mortar.

#### X-ray diffraction (XRD) analysis of biocement mortar

**Figure 4:**
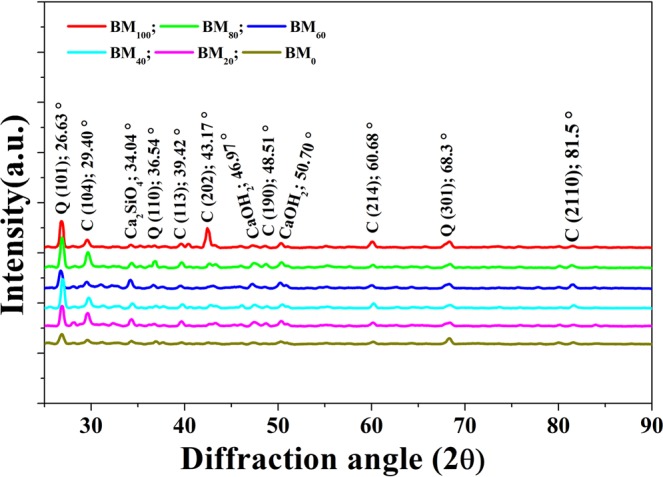
XRD spectrum of biocement mortar in different samples treated with different percentage (%) of wastewater (BM_0_, BM_20_, BM_40_, BM_60_, BM_80_ and BM_100_).

Figure 4 shows the of XRD result of the cement mortar. The quartz phase was observed the peak intensity at *2θ* for the value around 26.63°, and 68.3° representing the Miller index of (101) and (301), respectively. The peak intensity at *2θ* for the value around 29.399°, 39.42°, 43.17°, 60.68°, and 81.5° representing the Miller index of (104), (113), (202), (214), and (2110), respectively for the formation of calcite phase. The observed aragonite and calcite are the products of calcium carbonate, which are formed in biocement mortar, influences by microbial organic wastewater; and further confirmed of a white powder which is calcium carbonate. The calcite peak intensities of biocement mortar (Fig. 4) treated from BM_20_ to BM_100_ are noticed evidently higher compare to BM_O_, which indicates the addition of organic wastewater is relevant to changes the amount and crystallization form of calcium carbonate. In comparison of compression strength and water absorption, the present result confirms the calcium carbonate precipitation through a biochemical process in presence of urea, calcium nitrate and urease (which generated from microorganisms in wastewater). Furthermore, the precipitated calcium carbonate can fill the pores of cement mortar that formed during cement-hydration reactions.

#### Morphology of biocement mortar

**Figure 5:**
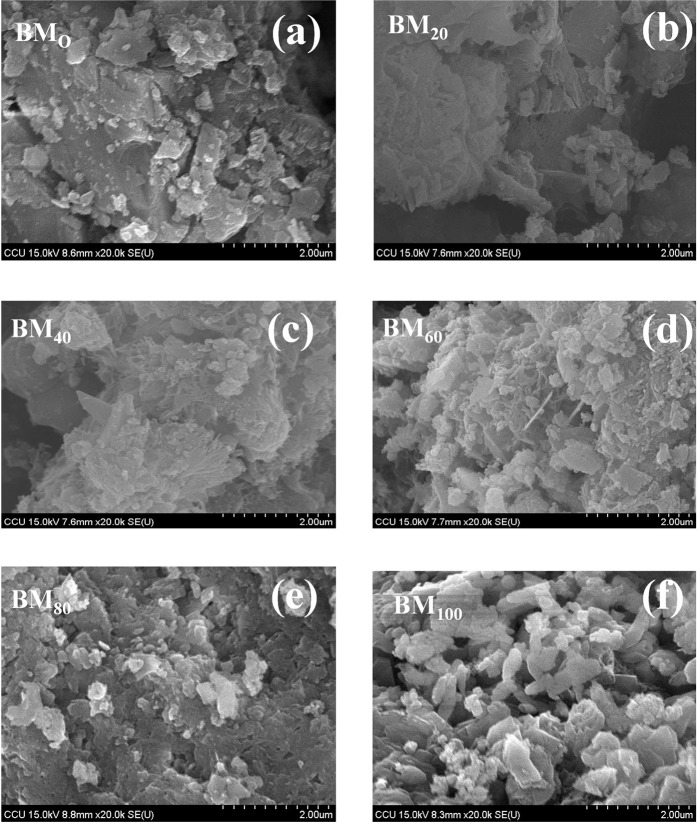
SEM micrograph of Biocement mortar in different samples treated with different percentage (%) of wastewater (BM_0_, BM_20_, BM_40_, BM_60_, BM_80_ and BM_100_).

**Figure 6:**
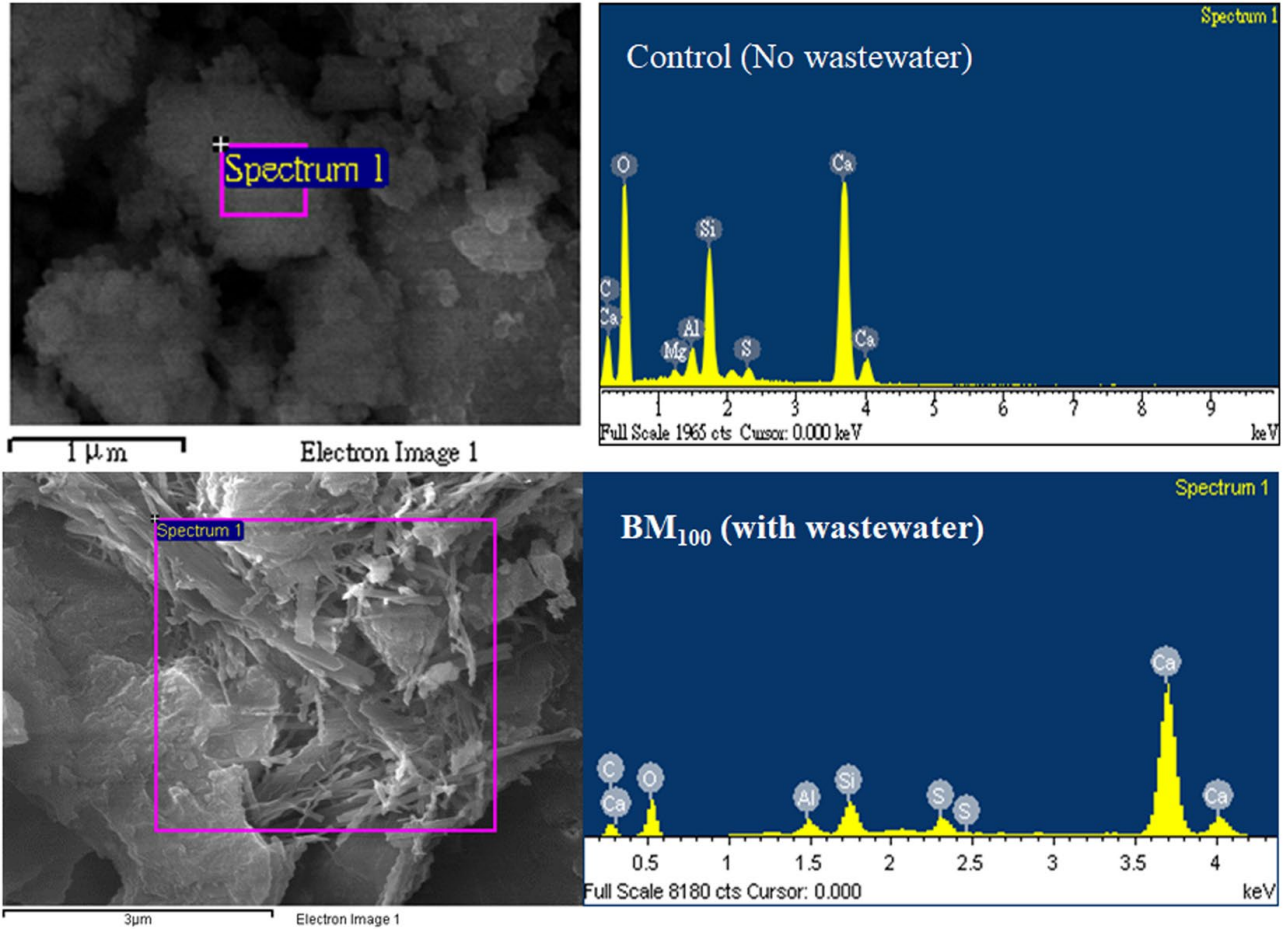
EDX micrograph: Formation and existence of CaCO_3_ in Bio-cement mortar: Control (no wastewater, only MiliQ water) and BM_100_ (with wastewater).

**Figure 7:**
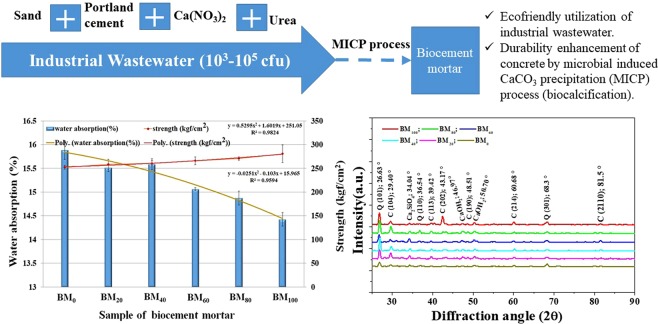
Schematic representation of sustainable management option of industrial wastewater resources in cement mortar application by microbial induced calcium carbonate precipitation process.

The morphological signature (SEM micrograph) of the biocement mortar containing 0% (BM_O_), 20% (BM_20_), 40% (BM_40_), 60% (BM_60_), 80% (BM_80_), and 100% (BM_100_) of wastewater are shown in Figs. 5 and 6. SEM-EDX micrograph shows the acicular, massive and stacked calcite structure in cement mortar; in particular, the needle shape, brick shape, and stacks of calcite crystals were observed inside cement mortar. Results shows at a higher proportion of waste water; the crystallization of calcite is more evident/pronounced. The most distinctive calcite crystallization is formed treated with 100% (BM_100_) waste water, where calcite crystals can fill the pores within the cement mortar to form the denser structure. This result can be mutually confirmed with the results of the strength and water absorption of cement mortar (see previous section). It is clear that the biologically produced calcite, precipitates within the concrete void and block pores/voids, thereby increasing the strength. Ghosh *et al*.^32^, reported that a *thermophilic* anaerobic microorganism increases the compressive strength of 25% in cement mortar in 28 days with the addition of about 10^5^ cell/ml of water. The strength improvement was reported due to growth of filler material within the pores of the cement–sand matrix by microbial growth and the process of microbiologically induced mineral precipitation^32^. In another research report, the *B. sphaericus* improves strength of cement concrete, where concrete-immobilized bacterial spores and able to seal the cracks by biomineral formation after being revived by water and growth nutrients^33^. The potential crack healing ureolytic bacteria (example *Bacillus sphaericus*,) are able to precipitate CaCO_3_ in their micro-environment by conversion of urea into ammonium and carbonate; as a results the cracks were filled completely^31^. Sujatha *et al*.^34^ reported a indigenous soil bacteria which enhance the compressive strength of cement mortar by precipitating the calcium carbonate mineral; as 18% of compressive strength was increased with 28 days, where the bacteria transformed soluble organic nutrients into insoluble inorganic calcite crystals (applicable for repair for concrete cracks). Hence, the present investigation reflects a positive direction of the application of microbial consortium of wastewater, which can be applicable and improve the strength, durability and repair of concrete cracks of cement concrete (Fig. 7).

## Conclusion

The industrial wastewater (10^3^–10^5^ cfu/ml) was applied to enhance the durability of biocement mortar such as compressive strength, water absorption by microbial-induced calcium carbonate precipitation (MICP) (biocalcification). The ‘strength’ of biocement mortar increased (R^2^ = 0.9825) and ‘water absorption’ of biocement mortar decreases (R^2^ = 0.9594) with the increasing percentage (%) of organic wastewater by MICP process. The highest ‘strength’ (280.75 kgf/cm^2^) and lower ‘water absorption’ (14.42%) was noticed in addition of 100% wastewater after 28 days. Morphological study reveals the acicular, massive and stacked calcite structure in cement mortar samples; in particular, the needle shape, brick shape, and stacks of calcite crystals were observed inside cement mortar. XRD analysis indicated the formation of calcium carbonate (aragonite and calcite) in biocement mortar which influences by hydrolysis of urea, catalyzes by microbial enzyme of urease in MICP process using microbial organic wastewater. The crystallization of calcite is more evident/pronounced in higher proportion of wastewater. The most distinctive calcite crystallization is formed in the samples of 100% (BM_100_) waste water, where calcite crystals fills the pores within the cement mortar to form the denser structure. Thus, the findings implied a cost-effective of MICP technology to improve the permeability of concrete and thereby enhancing the life of concrete structures along with the mitigation of industrial wastewater pollution, which also goes some way towards solving the problem of industrial wastewater pollution.

## Methods

### Characterization of industrial wastewater

The food industrial wastewater was collected from “Grape King Bio” company (wastewater release 8462 tons per month) and used to produce biocement mortar by MICP process. The basic wastewater parameter such as pH (HI 9828 Multiparameter, HANNA, Taiwan), COD (Chemical Oxygen Demand) (mg/l) (NOVA-60, MERCK), BOD (mg/l) (Biological Oxygen Demand) (NOVA-60, MERCK), Color (Pt-Co), TSS (mg/l) (Total suspended solids) (HI 9828 Multiparameter HANNA, Taiwan), temperature (°C) (HI 9828 Multiparameter, HANNA, Taiwan) and colony-forming unit (CFU) was measured during sample collection, and stored properly for further use. The urease activity was measured immediately after sampling following the procedure of Chen *et al*.^14^.

### Experimental procedure of MICP process

The urea, Ca(NO_3_)_2_ and food industrial wastewater were used for CaCO_3_ precipitation by MICP process. The schematic experimental conditions are shown in Table 2. Urea (final concentration 1.1 M) was mixed with different concentration of Ca(NO_3_)_2_ (0.1 M, 0.3 M, 0.5 M and 1.1 M) considering the final volume 40 ml by food industrial wastewater. Mixture was incubated for 24 h at 30 °C, with shaking at 120 rpm for precipitation. The precipitates were collected by centrifuging at 5000 rpm and dry at 50 °C for 3 days. The dry powder was weighted by gravimetric method and store for further study. The chemical character synthesized powder particle was measured by XRD analysis. The urease activity in wastewater was measured following the procedure of Chen *et al*.^14^.

### Preparation of biocement mortar

**Figure 8:**
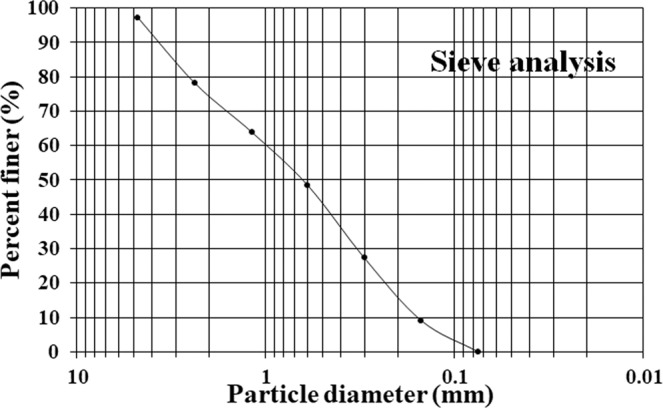
Grains size distribution chart of Sand.

A standard Portland cement (produced by “Taiwan Cement”; Type-Ι, specific weight: 3.15) (Table 3) and natural river sand (Table 4; Fig. 8) was used for biocement mortar experiment. Both of natural water (as control) and industrial wastewater was used for the formation of biocement mortar considering the ratio or proportion as 0.6 [water to cement (W/C)] (Table 5). Since the formation of pores in cement mortar by cement-hydration reactions are small to survive^8,31,35^ the microorganisms, it is necessary a significant larger pore size within the cement mortar for MICP process in building materials^5,8,31,35^. Therefore, in the present study, the river sand (<0.075 mm) (grains size distribution is shown in the Fig. 8) was used into the mortar to form larger pores that could improve the survival of microorganisms for MICP. The biocement mortar was prepared using fixed concentrations of urea and Ca(NO_3_)_2_ at 1.1 M (consider as per standardized results of the highest precipitation in MICP process from section “Experimental procedure of MICP process”), while 40% of river sand was used in mortar. The industrial wastewater was used in the range of 20–100% (with 20% interval), where a control experiment (BM_0_) was design with 100% natural water. The different composition of cubic shapes of biocement mortar (BM_0_, BM_20_, BM_40_, BM_60_, BM_80_ and BM_100_) were prepared to optimized the MICP within the biocement mortar cube. In each composition (as per Table 3) of biocement mortar, the Portland cement and natural sand was mixed with low speed (140 $\pm$ 5 rpm) for 1 min, and then the natural water and organic wastewater was mixed as well as stirred for 1.5 minutes before switched to medium speed (285 ± 10 rpm) for 1 min. The mixture was cast in a 125 cm^3^ (5 cm×5 cm×5 cm) cube mold for 24 hours with the water-cement ratio (W/C) of 0.6. After demolding, the cubic sample preserved for 28 days at 70 ± 2% RH and 20 °C ± 2 °C for further study. The urease activity of different percentage (%) of wastewater in different set of samples (BM_0_, BM_20_, BM_40_, BM_60_, BM_80_ and BM_100_) were measured following the procedure of Chen *et al*.^14^. Also, the urease activity of the mixer of cement and waste water was estimated. The porosity of the bicement mortar was measured following the procedure of Emamian and Eskandari-Naddaf^36^.

**Table 3:** Physical property of cement mortar (CNS 61 R2001, 2011; CNS 1078 R3039, 2011)^40,41^.

Parameter	Test results	Specification
	Type-Cement	Type-I Cement
		CNS 61;CNS 1078^40^
Specific weight (m^2^/kg)	3.15	—
Fineness (air permeability test) (m^2^/kg)	352	min.280
Initial set (min)	215	min.45
Final set (min)	286	max.375
Soundness (autoclave expansion test) (%)	1	max.0.80
Air content (specific mass) (%)	7.4	max.12
SiO_2_ (%)	20.21	
Fe_2_O_3_ (%)	2.97	
Al_2_O_3_ (%)	5.35	
CaO (%)	60.55	
MgO (%)	3.94	
SO_3_ (%)	2.51	
Loss on ignition (%)	1.3

**Table 4:** Physical property of natural river sand (CNS 486 A3005, 2015)^42^ (Fineness modulus = 2.76; Specific weight = 2.64; 24-hour water absorption − 0.9%).

Screen number	Size (mm)	Percentage of accumulated residue	Percentage of screening
#4	4.75	2.75	97.25
#8	2.36	21.94	78.06
#16	1.18	36.16	63.84
#30	0.6	51.51	48.49
#50	0.3	72.69	27.31
#100	0.15	90.91	9.09
#200	0.075	100.00	0.00

**Table 5:** Different experimental conditions (BM_O_ – BM_100_) in varied parameters for biocement mortar.

Type	BM_O_	BM_20_	BM_40_	BM_60_	BM_80_	BM_100_
Cement (g)	81.78	81.78	81.78	81.78	81.78	81.78
Water (g)	49.068	39.254	29.441	19.627	9.814	0
Waste water (g)	0	9.814	19.627	29.441	39.254	49.068
Sand 40% (g)	130	130	130	130	130	130
Urea (g)	3.24	3.24	3.24	3.24	3.24	3.24
Ca(NO_3_)_2_.4H_2_O (g)	12.74	12.74	12.74	12.74	12.74	12.74
W/C	0.6	0.6	0.6	0.6	0.6	0.6
Water	100%	80%	60%	40%	20%	0%
Wastewater	0%	20%	40%	60%	80%	100%

### Assessment of biocement mortar properties

#### Estimation of compressive strength of biocement mortar

The measurement of compressive strength of cubic biocement mortar (BM_0_, BM_20_, BM_40_, BM_60_, BM_80_ and BM_100_) are conducted according to CNS1010 R3032^37^. The measurement of cubic biocement mortar was conducted with 3 replicates; repeated for 3 times (YS/5001–25 T, YENSTRON, Taiwan). Center of the samples is placed in the compression testing machine for testing and the compression load is increased at a speed of 0.5 mm/min until the sample can no longer sustain the compression, and the structural integrity is damaged. To calculate the compressive strength of the sample, the maximum load was recorded, and divided by the cross-section area of the sample.

#### Estimation of water absorption of biocement mortar

The change of water absorption by CaCO_3_ precipitation that may occurs by the MICP process within the biocement mortar cube and fills the pores of the cement mortar samples. The water absorption test was conducted on cement mortar samples (BM_0_, BM_20_, BM_40_, BM_60_, BM_80_ and BM_100_) following the procedure of ASTM C642^38^. The measurement of water absorption of the biocement mortar cube was carried out by drying the biocement mortar cube to a constant temperature at 110 °C in an oven, and the gravimetric weights were measured at 24 h intervals until the mass balance between initial and final weight less than 0.5%. The dry biocement mortar cubewas then immersed in water at 21 °C for 48 h, and after taking out, the surface was wiped dry, and the mass of the saturated substance after the immersion was calculated.

The percentage of water adsorption was calculated as follow as. Water absorption (%) = (C-A)/(C-D) × 100; where A is the weight (g) of the oven dried sample in air; C is the weight (g) of sample after immersion and boiling; and D is the apparent weight (g) of sample in water after immersion and boiling.

#### Characterization of synthesized material

The crystallinity of MICP synthesized powders and biocement mortar cube was analyzed by XRD (Shimadzu XRD-6000) with CuKa radiation (λ = 0.15418 nm) at 40 kV and 30 mA. The angle was set to 20–80°, with two degrees (*2θ*) per minute. Morphological study of the biocement mortar cube particles was conducted by Field-Emission Scanning Electron Microscope (FE-SEM) analysis (TOPCONABT-150S, Japan) with a coating (Pt) operated at 0.1–30 kV.
